# Complete Genome Sequence of an *Edwardsiella piscicida*-Like Species, Recovered from Tilapia in the United States

**DOI:** 10.1128/genomeA.01004-15

**Published:** 2015-09-03

**Authors:** Stephen R. Reichley, Geoffrey C. Waldbieser, Mark L. Lawrence, Matt J. Griffin

**Affiliations:** a College of Veterinary Medicine, Mississippi State University, Starkville, Mississippi, USA; b USDA-ARS Warmwater Aquaculture Center, Stoneville, Mississippi, USA; c College of Veterinary Medicine, Mississippi State University, Stoneville, Mississippi, USA

## Abstract

An *Edwardsiella piscicida*-like species is a Gram-negative facultative anaerobe that causes disease in some fish species. In this report, we present the complete and annotated genome of isolate LADL05-105, recovered from cultured tilapia reared in Louisiana, which contains a chromosome of 4,142,037 bp and no plasmids.

## GENOME ANNOUNCEMENT

The genus *Edwardsiella*, first recognized in the 1960s (1), is a diverse group of Gram-negative bacteria that infect a range of mammalian, avian, reptilian, and fish hosts worldwide (2). Until recently, the genus consisted of three taxa: *E. hoshinae*, *E. ictaluri*, and *E. tarda* (3). Research into the heterogeneity of *E. tarda* utilizing multilocus sequence analysis, comparative phylogenomic analysis, phenotypic characterization, and DNA-DNA hybridization identified three genetically distinct taxa, resulting in the adoption of *E. piscicida* and the identification of a group of isolates presently referred to as *E. piscicida*-like species (3–6). In 2015, an isolate from this *E. piscicida*-like sp. group (*Edwardsiella* strain 080813) was designated the type strain of the recently proposed taxon, *E. anguillarum* sp. nov. (7). Furthermore, research has shown an apparent difference in mortality in channel catfish infected with *E. tarda*, *E. piscicida*, and *E. piscicida*-like species (8).

Genomic DNA from *E. piscicida*-like sp. isolate LADL05-105 was sequenced using two method, Illumina (36× coverage) and Pacific Biosciences (Pac-Bio) (113× coverage). The PBcR module of wgs-8.2beta (Celera) (9, 10) was used to identify the longest 40× coverage Pac-Bio reads, which were corrected with the remaining Pac-Bio data. The longest 25× coverage of corrected sequence was assembled into a single, circular chromosome. Illumina sequencing reads were mapped to the assembled chromosome with Bowtie2 (11) to correct variations in homopolymer lengths and the consensus sequence was produced using VarScan version 2.3.7 (12).

The circularized and completed genome was submitted to the National Center for Biotechnology Information Prokaryotic Genomes Annotation Pipeline (PGAP) for annotation and submission to GenBank. The genome data was also subjected to RAST (13, 14) annotation using the Glimmer option.

This *E. piscicida*-like species genome consists of one circular chromosome with 4,142,037 bp and 58.8% GC content. PGAP annotation predicted 3,686 genes encoding 3,159 proteins and 99 tRNAs. RNAmmer (15) predicted 9 rRNA operons. Average nucleotide identities (ANI), as calculated by Goris et al. (16), were estimated using an online calculator (http://enve-omics.ce.gatech.edu/ani). The complete genome of LADL05-105 shares an ANI of 99.84% with *Edwardsiella* strain 080813, which was recently proposed as the type strain for *Edwardsiella anguillarum* sp. nov. (GenBank accession no. CP006664) (5, 7). The isolate also shares 99.59% ANI with *E. piscicida*-like sp. isolate EA181011 (GenBank accession no. CP011364) (17), 94.39% with *E. piscicida* isolate C07-087 (GenBank accession no. CP004141) (18), 92.48% with *E. ictaluri* isolate 93-146 (GenBank accession no. CP001600) (19), and 84.01% with *E. tarda* isolate FL95-01 (GenBank accession no. CP011359) (20). These ANI values suggest that isolates LADL05-105, 080813, and EA181011 are representatives of the same *Edwardsiella* species. RAST analysis revealed 150 unique subsystems in LADL05-105 compared to *E. tarda* isolate FL95-01, including elements of inositol catabolism, mannitol utilization, and type VI secretion system. Compared to *E. piscicida* isolate C07-087, RAST analysis showed that LADL05-105 contained 108 unique subsystems, including elements involved in mannitol utilization and copper hemostasis. The LADL05-105 genome does not carry any plasmids.

### Nucleotide sequence accession number. 

The complete genome sequence for *Edwardsiella piscicida*-like sp. isolate LADL05-105 has been deposited in GenBank under the accession number CP011516.
